# Efficacy of Unrelated Allogeneic Hematopoietic Stem Cell Transplantation in Severe Aplastic Anemia: A Single-Center Retrospective Study

**DOI:** 10.7150/ijms.137416

**Published:** 2026-09-11

**Authors:** Jinrong Zhao, Liangliang Wu, Ming Zhou, Xiaowei Chen, Wenjian Mo, Ruiqing Zhou, Yumiao Li, Caixia Wang, Shilin Xu, Shiyi Pan, Wei Zhou, Tingfen Deng, Yuping Zhang, Shunqing Wang

**Affiliations:** Department of Hematology, Guangzhou First People's Hospital, South China University of Technology, Guangzhou, Guangdong, China; Department of Hematology, Guangzhou First People's Hospital, Guangzhou Medical University, Guangzhou, Guangdong, China.

**Keywords:** SAA, HSCT, URD, OS, aplastic anemia

## Abstract

To evaluate the efficacy of unrelated allogeneic hematopoietic stem cell transplantation (URD-HSCT) in severe aplastic anemia (SAA), this study retrospectively analyzed the clinical data of 206 patients at a single center. The median age was 28 years (range: 5-64 years), and 13.6% had received immunosuppressive therapy prior to transplantation. The median time to neutrophil and platelet engraftment was 12 days (range: 9-22 days / 8-55 days), respectively, with a graft failure rate of 3.4%. The cumulative incidence of grade II-IV acute graft-versus-host disease (aGVHD) was 21.7% (95% CI: 16.6%-27.8%), and that of chronic GVHD was 20.4% (95% CI: 15.4%-26.6%). Survival analysis revealed a 7-year overall survival (OS) rate of 91% for the entire cohort. Notably, the 7-year OS rate was significantly higher in the donor age ≤30 years group compared to the >30 years group (97% vs 85%, *P* = 0.0028), and in the HLA 10/10 matched group compared to the 9/10 matched group (95% vs 86%, *P* = 0.023). Multivariate analysis identified donor age >30 years, and HLA 9/10 matching as independent risk factors for OS and graft-versus-host disease-free, relapse-free survival (GRFS). URD-HSCT is an effective treatment for SAA, and selecting an appropriate donor can improve treatment outcomes.

## Introduction

Severe aplastic anemia (SAA) is an immune-mediated bone marrow failure disorder characterized by anemia, bleeding, infections, and pancytopenia. The main treatment options include immune-suppressive therapy (IST) and allogeneic hematopoietic stem cell transplantation (HSCT)^1^, ^2^. Standard IST (ATG+CsA) achieves an initial response rate of approximately 60-65%. In long-term follow-up, clinical management involves addressing potential relapse and monitoring for rare instances of clonal progression toward MDS or AML, both of which may influence long-term prognosis^3^-^5^.

Matched sibling donor hematopoietic stem cell transplantation (MSD-HSCT) is the preferred treatment option for patients with SAA, achieving an overall survival rate of 83% to 90%^6^, ^7^. However, this type of donor is available in only about 30% of cases^8^. In addition, with ongoing efforts such as improvements in conditioning regimens and adjustments in graft-versus-host disease (GVHD) prophylaxis, haploidentical hematopoietic stem cell transplantation (haplo-HSCT) has been shown to yield favorable outcomes in patients with SAA or very severe aplastic anemia (VSAA) who are refractory to immunosuppressive therapy, and its indications have been further expanded^9^, ^10^. Unrelated hematopoietic stem cell transplantation (URD-HSCT) also represents a viable alternative; however, it necessitates careful consideration of challenges such as donor search difficulties, extended waiting periods, and transplant-associated risks, including severe GVHD^11^. However, a previously published multicenter retrospective study indicated that for SAA patients without MSD, URD-HSCT can achieve better FFS (failure-free survival) and GFFS (graft failure-free survival) compared to IST+EPAG^12^. More comprehensive research is needed to understand the current efficacy of URD-HSCT in SAA patients.

Here, we report the results of a retrospective analysis of 206 cases of unrelated allogeneic hematopoietic stem cell transplantation performed at our center for patients with SAA over the past 13 years (from November 2012 to December 2024).

## Methods

### Patients

This study conducted a retrospective analysis of 206 patients with SAA who underwent HSCT from unrelated donors at the Department of Hematology, Guangzhou First People's Hospital, between November 2012 and December 2024. All patients included in this study were diagnosed with SAA according to established criteria^13^, ^14^. To be eligible, individuals had to lack an accessible matched sibling donor (MSD). Those with inherited bone marrow (BM) failure syndromes, as well as severe aplastic anemia (SAA) patients presenting with active paroxysmal nocturnal hemoglobinuria-characterized by persistent hemolysis and/or thrombotic events-were not included in the study. Patients were followed up through the electronic medical record system or by telephone, with the last follow-up date being September 30, 2025. This study was approved by the Ethics Committee of Guangzhou First People's Hospital and was conducted in accordance with the Declaration of Helsinki. Written informed consent was obtained from all patients or their guardians prior to treatment.

### Transplantation procedures and definitions

All transplant procedures followed established protocols for conditioning regimens, stem cell collection, and supportive care as previously described in the literature^12^, ^15^, ^16^. In clinical practice, when multiple HLA-matched unrelated donors were available, the following hierarchy was applied: (1) younger donor; (2) male sex preference; (3) non-cytomegalovirus (CMV) seropositivity in CMV-negative recipients. Three distinct conditioning regimens were employed: (1) Flu/Cy/ATG, comprising fludarabine (30 mg/m²/day, days -7 to -4), cyclophosphamide (40 mg/kg/day, days -7 to -4), and rabbit ATG (2.5 mg/kg/day, days -5 to -2); (2) Bu/Cy/ATG, featuring busulfan (0.8 mg/kg qid, days -7 to -6), cyclophosphamide (50 mg/kg/day, days -5 to -2), and rabbit ATG (2.5 mg/kg/day, days -5 to -2); and (3) a modified PTCy (mPTCy) protocol utilizing augmented ATG dosing (2.0 mg/kg/day, days -5 to -3) and attenuated post-transplant cyclophosphamide (40 mg/kg/day, days +3 and +4). Mobilized peripheral blood stem cells (PBSCs) served as the sole source of hematopoietic stem cells. Cyclosporine A (CSA) was delivered intravenously at a dosage of 1.5 mg/kg every 12 hours beginning on day +3, with a target trough level of 150-250 ng/mL; once bowel function recovered, the regimen was switched to an oral preparation. Trough concentrations of CSA were sustained for up to 12 months after transplantation, after which the dose was gradually reduced over a 6-month period. Mycophenolate mofetil (MMF) was started on day -7 at an oral dose of 0.5 g every 12 hours (adjusted to 0.25 g every 12 hours for pediatric recipients), reduced to one-half of the initial dose on day +30, and then ceased entirely on day +45. Methotrexate (MTX) was administered intravenously at 15 mg/m² on day +1, with subsequent doses of 10 mg/m² given on days +3, +6, and +11. Neutrophil engraftment was defined as the first of three consecutive days with an absolute neutrophil count (ANC) ≥0.5×10⁹/L. Platelet engraftment was defined as the first of seven consecutive days with a platelet count ≥20×10⁹/L without transfusion support. Primary graft failure was defined as failure to achieve an ANC ≥0.5×10⁹/L by day 28 post-transplantation and confirmed donor-derived hematopoiesis failure. Secondary graft failure was defined as subsequent pancytopenia, bone marrow hypoplasia, loss of donor chimerism, and absence of moderate-to-severe chronic GVHD after initial successful engraftment^17^. Acute GVHD (aGVHD) and chronic GVHD (cGVHD) were diagnosed and graded according to published international criteria^18^, ^19^. Overall survival (OS) was calculated from the date of transplantation to death from any cause or the last follow-up. Graft-versus-host disease-free, relapse-free survival (GRFS) was defined as the time free from disease recurrence or death, and free from grade III-IV aGVHD or extensive cGVHD.

### Statistical analysis

Statistical analysis and graphing were performed using R version 4.1.3 and GraphPad Prism 9.0. Continuous variables are presented as medians (ranges), and categorical variables are expressed as counts and proportions. OS and GRFS were estimated using the Kaplan-Meier method and compared using the log-rank test. The cumulative incidences (CuIs) of aGVHD and cGVHD were calculated via competing risks analysis employing Gray's test, where death from any etiology served as the competing event. Multivariate survival analysis was conducted using Cox regression analysis. A P value < 0.05 (two-tailed) was considered statistically significant.

## Results

### Patient characteristics

A total of 206 patients with severe aplastic anemia were included in this study. Among them, 110 patients (53.4%) were male, and the median age was 28 years (range: 5-64 years). Forty-six patients (22.3%) met the criteria for very severe aplastic anemia (VSAA). Twenty-eight patients (13.6%) had received immunosuppressive therapy prior to transplantation. Forty-six patients (22.3%) had paroxysmal nocturnal hemoglobinuria (PNH) clones. Regarding HLA matching, the numbers of patients with 10/10 and 9/10 matches were similar (115 vs. 91, respectively). 126 patients (61.2%) had blood types that were mismatched with their donors. Most donors were male (173 out of 206, 84%). Detailed patient characteristics are shown in **Table 1**.

### Engraftment

Out of the 206 patients, 202 achieved successful hematopoietic reconstitution. The median time to neutrophil engraftment was 12 days (range: 9-22 days), and the median time to platelet engraftment was also 12 days (range: 8-55 days). Graft failure occurred in 7 patients (7/206, 3.4%). Among them, two patients experienced primary graft failure. One patient died from Scedosporium apiospermum sepsis before engraftment, and another died from heart failure and severe pneumonia. In addition, five patients developed secondary graft failure. One of these patients achieved engraftment after receiving an umbilical cord blood transfusion following the secondary graft failure. Another patient died from cytomegalovirus (CMV) pneumonia. Three patients who experienced secondary graft failure subsequently underwent a second transplant.

### Immune reconstitution

In this study, we evaluated the numbers of lymphocytes and immunoglobulins at 1, 2, 3, 6, and 12 months after HSCT. NK cells recovered the fastest and consistently remained within the normal range (Figure 1D). Adaptive immune T lymphocytes recovered earlier than B lymphocytes. CD3+ T cells approached normal levels by the third month post-transplantation and continued to increase (Figure 1A). Moreover, the recovery of CD8+ cytotoxic T cells was significantly faster than that of CD4+ helper T cells. CD8+ T cells returned to normal levels by the second month, whereas CD4+ T cells remained below normal levels throughout the 12-month post-transplantation period (Figure 1B-C). Similarly, CD19+ B cells dropped close to zero during the first three months after transplantation, then began to recover slowly and reached normal levels by the 12th month (Figure 1E).

Regarding immunoglobulins, the concentrations of IgG (Figure 1G) and IgM (Figure 1H) showed some degree of fluctuation but remained within the normal range. In contrast, the concentration of IgA (Figure 1F) remained low during the first six months and normalized within the first year after HSCT.

### Infections

Post-transplantation, CMV viremia occurred in 123 patients (59.7%). In December 2021, letermovir was widely introduced in China for CMV prophylaxis in patients undergoing allo-HSCT. Using this as a clinical milestone, the incidence of CMV infection among SAA patients was 71.5% (98/137) prior to December 2021, compared to 36.2% (25/69) after January 2022. Among them, 16 patients (16/206, 7.8%) developed cytomegalovirus retinitis, 3 had cytomegalovirus enteritis, 1 had cytomegalovirus encephalitis, and 1 had cytomegalovirus pneumonia. Epstein-Barr virus (EBV) viremia occurred in 29 patients (14.1%). Of these, 10 patients (10/206, 4.9%) developed Epstein-Barr virus-associated lymphoproliferative disorders (EBV-LPD).

### GVHD

Excluding patients who died early, the cumulative incidence of aGVHD within 100 days post-transplantation was 26.6% (95% CI: 21%-33.1%) (Figure 2A), among which the cumulative incidence of grade II-IV aGVHD was 21.7% (95% CI: 16.6%-27.8%). A total of 196 patients in the cohort survived beyond 100 days and were therefore eligible for cGVHD assessment. The cumulative incidence of cGVHD was 20.4% (95% CI: 15.4%-26.6%) (Figure 2B), with the cumulative incidence of moderate to severe cGVHD being 8.2% (95% CI: 5.1%-12.9%). We then compared the incidence of aGVHD and cGVHD among different conditioning regimens. The cumulative incidence of aGVHD was higher with the Bu/Cy/ATG regimen (n = 48) than with the Flu/Cy/ATG regimen (n = 71), and the lowest with the mPTCy regimen (n = 87), although the differences were not statistically significant (*P* = 0.23) (Figure 2C). Similarly, there were no statistically significant differences in the incidence of cGVHD among the different conditioning regimens (*P* = 0.61) (Figure 2D).

### Survival

The median follow-up time for all patients was 56.6 months (range: 0.4-156.9 months). A total of 8.3% (17/206) of patients died, with infection being the leading cause of death (11/17, 64.7%).

The 7-year overall survival (OS) in our patient cohort was 91% (Figure 3A). For patients aged ≤30 years, the 7-year OS was 94%, compared to 88% for those older than 30 years (*P* = 0.15) (Figure 3B). Patients diagnosed with very severe aplastic anemia had a 7-year OS of 89%, while those with non-VSAA had a 7-year OS of 92%; the difference was not statistically significant (Figure 3C). Similarly, there was no significant difference in 7-year OS between patients who underwent transplantation ≤ 6 months after diagnosis and those who waited more than 6 months (both 91%) (Figure 3D). Further subgroup analyses using cut-off intervals of 5 years and 10 years from diagnosis to transplantation also showed no statistically significant differences. Among patients who had received immunosuppressive therapy prior to transplantation, the 7-year OS was 96%, compared to 91% for those who had not received IST (*P* = 0.37) (Figure 3E). For patients with a Hematopoietic Cell Transplantation-Comorbidity Index (HCT-CI) score of 0-1, the 7-year OS was 91%, whereas for those with a score ≥ 2, it was 90% (*P* = 0.87) (Figure 3F). Regarding donor characteristics, as the median donor age of this cohort was 30 years (range from 20 to 50 years), we used this as a cutoff for survival analysis. Patients with donors aged ≤ 30 years achieved a significantly higher 7-year OS of 97%, whereas those with donors aged > 30 years had a 7-year OS of 85% (*P* = 0.0028) (Figure 3G). In terms of HLA matching, patients with a 10/10 HLA-matched donor had a 7-year OS of 95%, whereas those with a 9/10 HLA-matched donor had a 7-year OS of 86%-a statistically significant difference (*P* = 0.023) (Figure 3H).

The 7-year graft-versus-host disease-free and relapse-free survival (GRFS) in our patient cohort was 79% (Figure 4A). For patients aged ≤ 30 years, the 7-year GRFS was 84%, compared to 73% for those older than 30 years (*P* = 0.07) (Figure 4B). Patients diagnosed with very severe aplastic anemia (VSAA) had a 7-year GRFS of 71%, while those with non-VSAA had a 7-year GRFS of 82%; the difference was not statistically significant (Figure 4C). Similarly, the 7-year GRFS was 80% for patients who underwent transplantation ≤ 6 months after diagnosis and 79% for those who waited more than 6 months (Figure 4D). Further subgroup analyses using diagnostic-to-transplant time cut-offs of 5 years and 10 years also showed no statistically significant differences. Among patients who had received immunosuppressive therapy (IST) prior to transplantation, the 7-year GRFS was 71%, compared to 81% for those who had not received IST (*P* = 0.31) (Figure 4E). For patients with a Hematopoietic Cell Transplantation-Comorbidity Index (HCT-CI) score of 0-1, the 7-year GRFS was 79%, whereas for those with a score ≥ 2, it was 90% (*P* = 0.43) (Figure 4F). Regarding donor characteristics, when the donor was aged ≤ 30 years, the 7-year GRFS was 86%, while for donors older than 30 years, the 7-year GRFS was 72% (*P* = 0.013) (Figure 4G). In terms of HLA matching, patients with a 10/10 HLA-matched donor had a 7-year GRFS of 86%, whereas those with a 9/10 HLA-matched donor had a 7-year GRFS of 71%-a statistically significant difference (*P* = 0.0089) (Figure 4H).

To identify an optimal donor profile, we further stratified patients into two groups: one consisting of donors aged ≤ 30 years with HLA 10/10 matching, and the other comprising all remaining patients. Survival analysis showed that the 7-year overall survival was 98% in the optimal donor group (≤ 30 years, 10/10 match) versus 88% in the other group (*P* = 0.023) (Figure 5A), while the 7-year GRFS was 86% versus 77%, respectively (*P* = 0.11) (Figure 5B). We compared the baseline clinical characteristics between the two groups of patients and found no significant differences in any factors except for the interval between diagnosis and transplantation (**Supplementary table 1**).

### Multivariate Analysis

We subsequently performed a multivariate analysis to identify independent risk factors affecting survival. The results showed that donor age greater than 30 years, and HLA 9/10 matching between donor and recipient were independent risk factors for overall survival. Similarly, donor age greater than 30 years and HLA 9/10 matching were also independent risk factors for graft-versus-host disease and relapse-free survival. Detailed results are shown in **Table 2**.

## Discussion

Unrelated allogeneic hematopoietic stem cell transplantation is an important treatment option for severe aplastic anemia, particularly for patients without a fully matched related donor. This study, based on long-term follow-up data, demonstrates that URD-HSCT has shown favorable efficacy in the treatment of SAA and provides evidence-based guidance for optimizing donor selection strategies.

In this study, the median time to neutrophil and platelet engraftment was 12 days, which is comparable to or even better than previously reported engraftment efficiencies in URD-HSCT (typically ranging from 10 to 21 days in the literature)^20^-^24^. Additionally, graft failure, as a serious complication of hematopoietic stem cell transplantation in SAA, still occurs at a rate of around 10% in general^25^-^28^, however, the rate in our center was only 3.4% (7/206). Given that SAA is a non-malignant disease, actively controlling the occurrence and progression of graft-versus-host disease remains a major challenge in URD-HSCT. Previous studies have reported that the incidence of aGVHD in patients receiving transplants from matched sibling donors or haploidentical donors can range from 30.3% to 35.4%. Our center's data showed an overall aGVHD incidence of 26.6%, with only one patient developing grade IV aGVHD. Furthermore, although the incidence of cGVHD in our cohort was higher than that observed in patients receiving transplants from matched sibling donors, it remained lower than that seen in patients undergoing haploidentical hematopoietic stem cell transplantation^6^, ^29^-^31^. In addition, the data on immune reconstitution also showed results similar to those from other research centers^6^, ^24^, ^29^. Overall, the high engraftment success rate and the controllable incidence of GVHD indicate that the URD-HSCT technical system in our center is well-established and largely consistent with the experience reported by both domestic and international transplant centers.

Achieving long-term survival is a key goal for patients with SAA undergoing HSCT. Our data show that the 7-year overall survival rate was 91%, which appears favorable when compared with historical outcomes of transplants from matched sibling donors or haploidentical donors. However, these cross-study observations should be interpreted with caution given the non-comparative, single-center design of the present study^6^, ^29^-^31^. With advancements in supportive care, enhanced anti-infection treatments, optimized conditioning regimens, and the availability of diverse GVHD prophylactic agents, factors previously considered to significantly impact prognosis have gradually evolved. For example, in our center, variables such as the interval between diagnosis and transplantation^32^, patient age, and HCT-CI^33^ were not found to influence survival outcomes^34^.

This study found that the 7-year OS rate was significantly higher in the donor age ≤ 30 years group compared to the > 30 years group. Furthermore, in the combined analysis, the “donor ≤ 30 years + HLA 10/10” group achieved an outstanding 7-year OS of 98%. A similar trend was also observed for GRFS, further highlighting the critical impact of donor age. These findings are consistent with previous studies in both hematologic malignancies and aplastic anemia, which have shown that hematopoietic stem cells from younger donors possess stronger proliferative potential and superior immunomodulatory capabilities. Transplants from younger donors are associated with faster engraftment, better immune reconstitution, and potentially reduced severity of GVHD^33^, ^35^-^37^. In addition, the 10/10 HLA-matched group demonstrated significantly higher 7-year OS and GRFS compared to the 9/10 HLA-matched group^38^. Multivariate analysis further confirmed that HLA 9/10 matching is an independent risk factor for both OS and GRFS. High HLA compatibility significantly reduces the risks of immune rejection (such as graft failure) and GVHD, underscoring the importance of selecting an appropriately matched unrelated donor.

This study is a single-center retrospective analysis, which may be subject to selection bias-for example, donor selection may be constrained by the actual availability within the donor registry. Three conditioning regimens were employed during the study period (Flu/Cy/ATG, Bu/Cy/ATG, and mPTCy), with their relative use evolving over time. In parallel, supportive care improved progressively, including updated antimicrobial prophylaxis and the introduction of letermovir for CMV prevention in December 2021. These calendar-period effects represent an inherent limitation of this retrospective, single-center study and may have independently contributed to the observed survival outcomes. Future research should involve multicenter prospective studies to further validate these findings and explore the synergistic effects of other donor characteristics, such as donor sex and CMV serostatus matching.

In summary, unrelated allogeneic hematopoietic stem cell transplantation is an effective curative treatment for severe aplastic anemia, and its efficacy is closely associated with donor characteristics. This study demonstrates that selecting an unrelated donor aged ≤ 30 years with HLA 10/10 matching can significantly improve long-term patient survival. In contrast, donor age >30 years and HLA 9/10 matching are identified as independent risk factors for OS. In clinical practice, such optimal donors should be prioritized to enhance the transplant outcomes of SAA patients.

## Supplementary Material

Supplementary table.

## Figures and Tables

**Figure 1:**
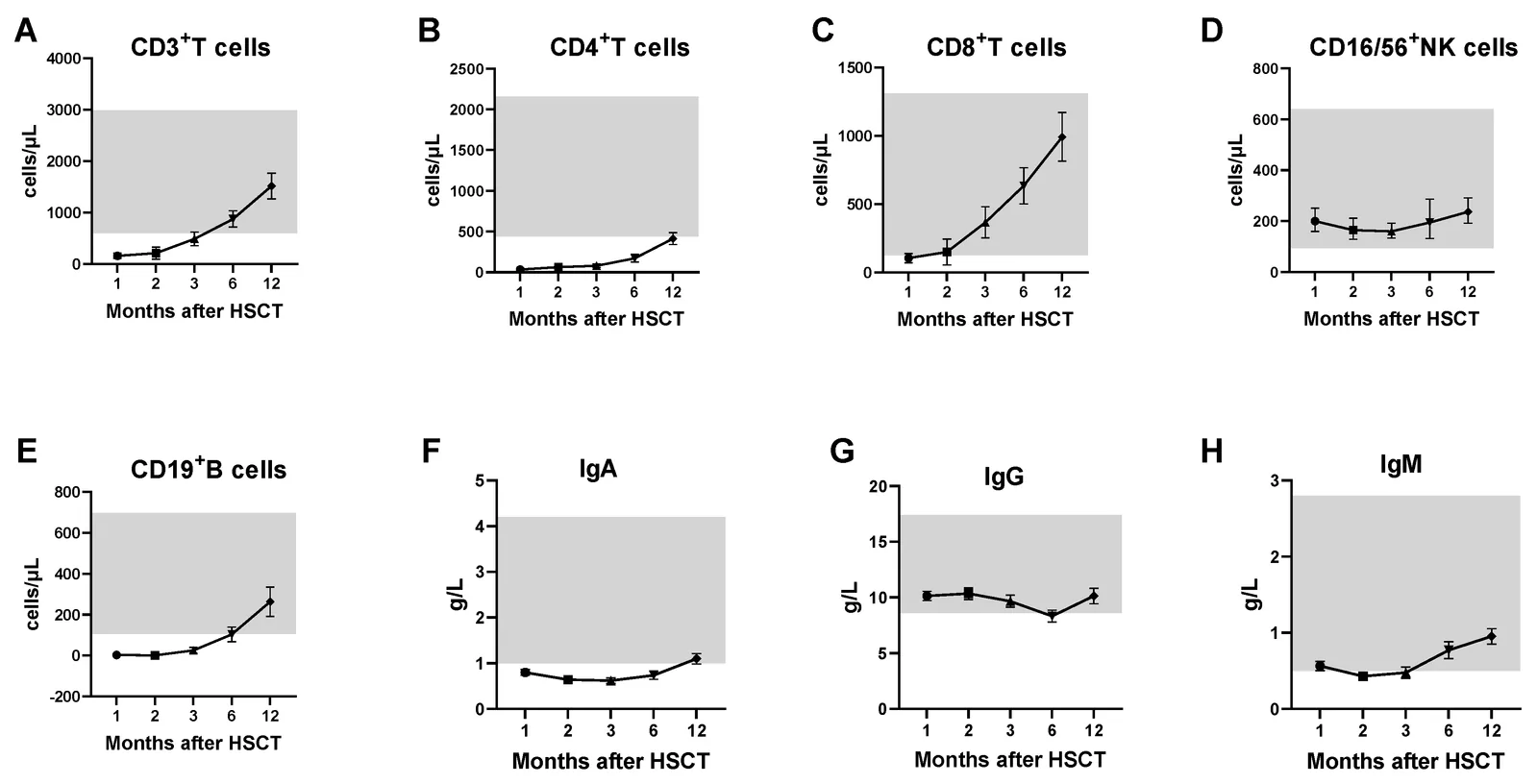
** The immune reconstitution after URD-HSCT in SAA patients.** Immune reconstitution in lymphocyte subsets (A-E) and immunoglobulins (F-H). Error bars indicate the 25th to 75th percentiles. Shaded areas represent the normal range.

**Figure 2:**
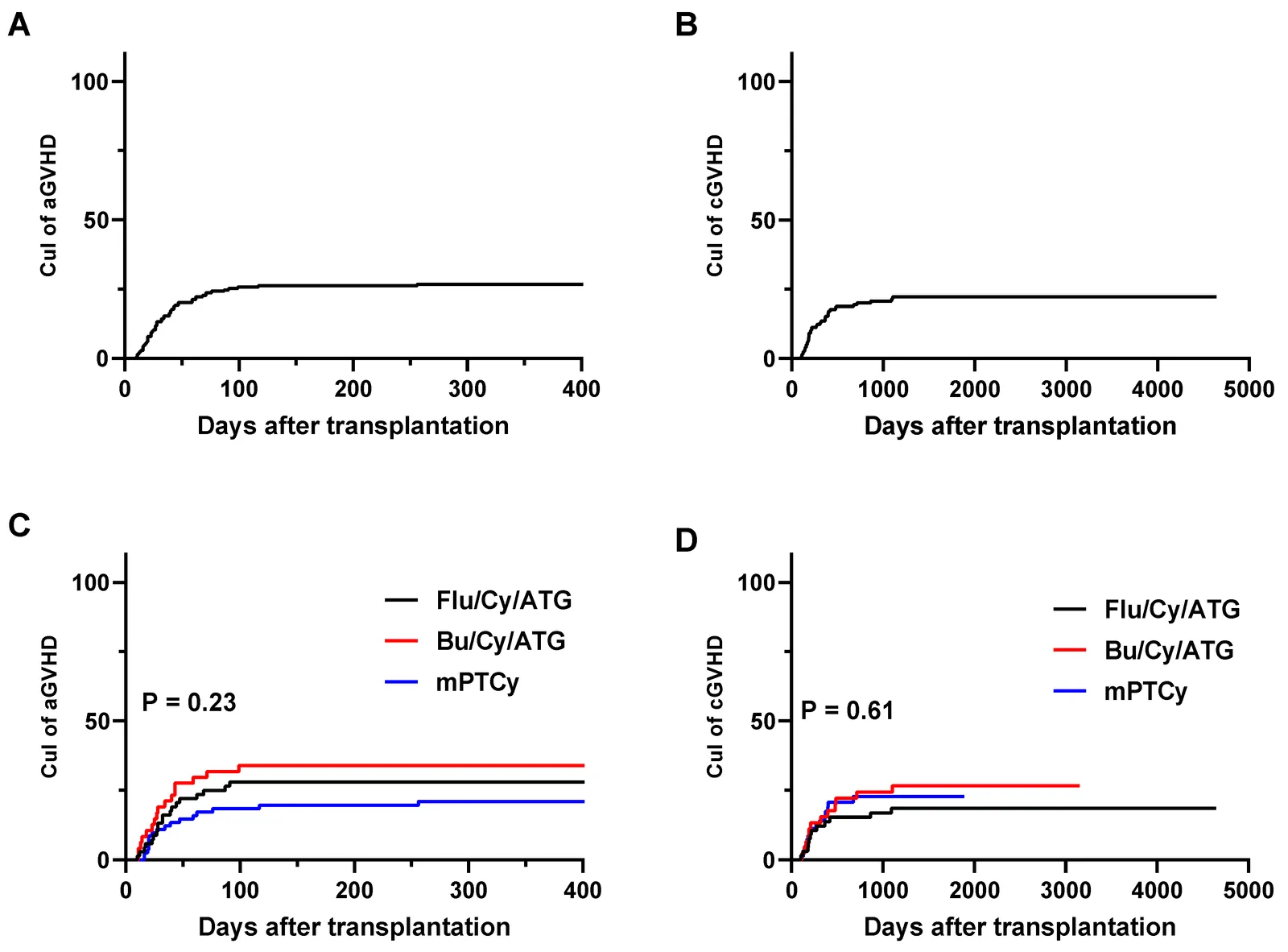
** GVHD outcomes in SAA patients who underwent URD-HSCT.** (A) CuI of aGVHD. (B) CuI of cGVHD. (C) CUI of aGVHD according to different conditioning regimens. (D) CUI of cGVHD according to different conditioning regimens.

**Figure 3:**
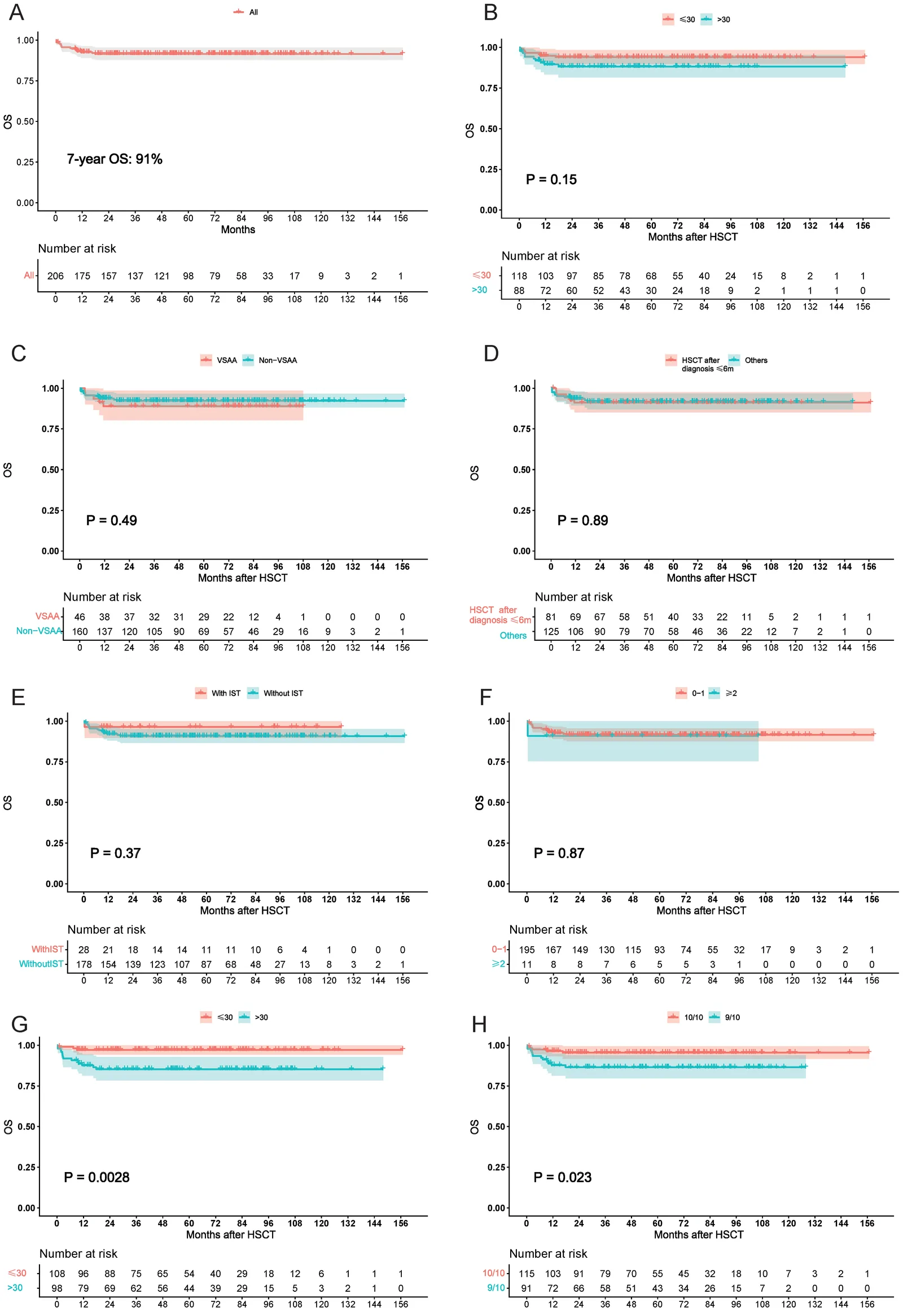
** Overall survival (OS) in patients with SAA.** (A) Overall survival of the entire cohort. (B) Overall survival according to different age groups. (C) Overall survival in patients with very severe aplastic anemia (VSAA) versus non-very severe aplastic anemia (non-VSAA). (D) Overall survival based on different intervals from diagnosis to transplantation. (E) Overall survival in patients who had previously received immunosuppressive therapy (IST) versus those who had not. (F) Overall survival according to different Hematopoietic Cell Transplantation-Comorbidity Index (HCT-CI) scores. (G) Overall survival based on different donor ages. (H) Overall survival according to different degrees of HLA matching.

**Figure 4:**
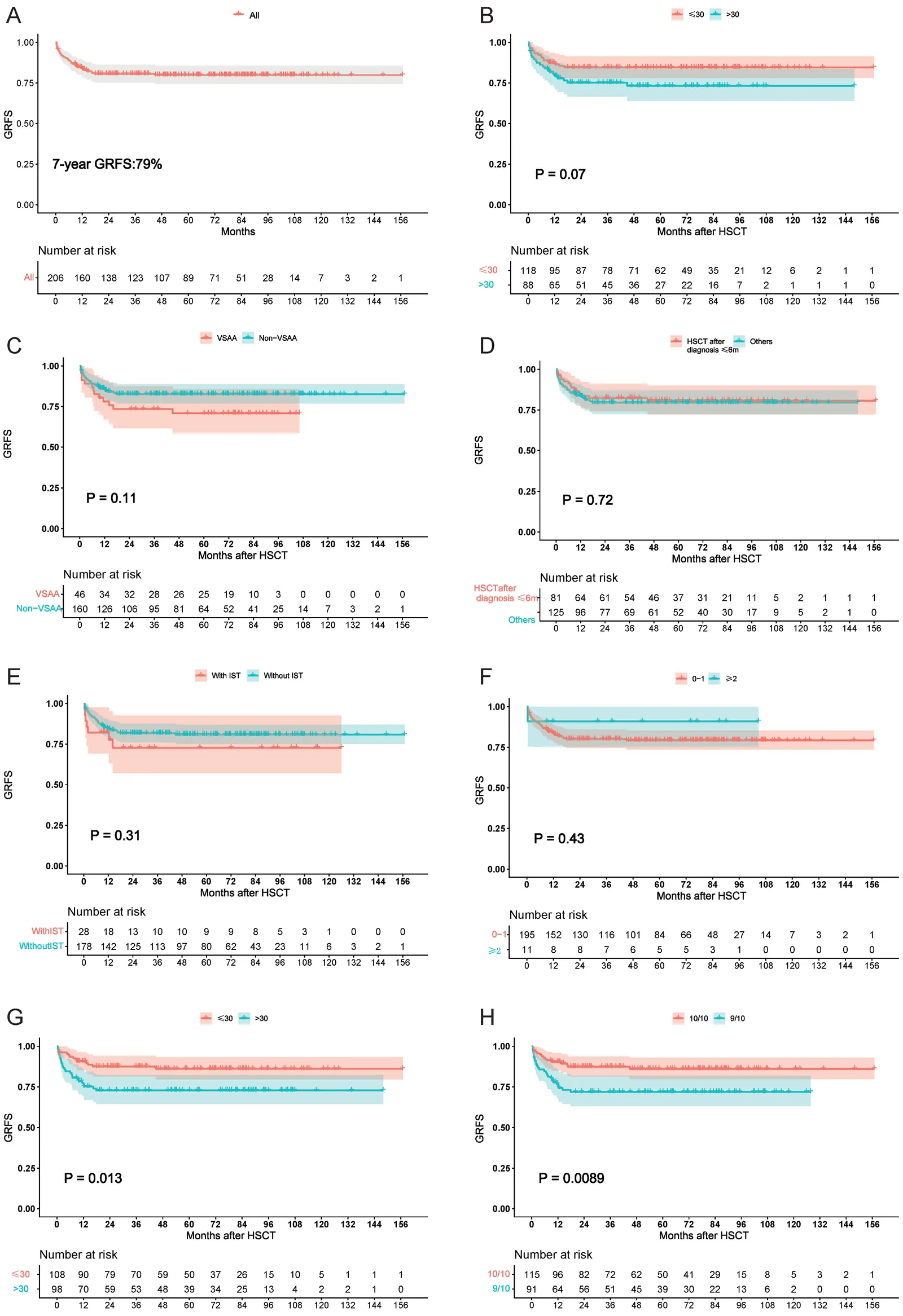
** Graft-versus-host disease-free, relapse-free survival (GRFS) in patients with SAA.** (A) GRFS of the entire cohort. (B) GRFS according to different age groups. (C) GRFS in patients with very severe aplastic anemia (VSAA) versus non-very severe aplastic anemia (non-VSAA). (D) GRFS based on different intervals from diagnosis to transplantation. (E) GRFS in patients who had previously received immunosuppressive therapy (IST) versus those who had not. (F) GRFS according to different Hematopoietic Cell Transplantation-Comorbidity Index (HCT-CI) scores. (G) GRFS based on different donor ages. (H) GRFS according to different degrees of HLA matching.

**Figure 5:**
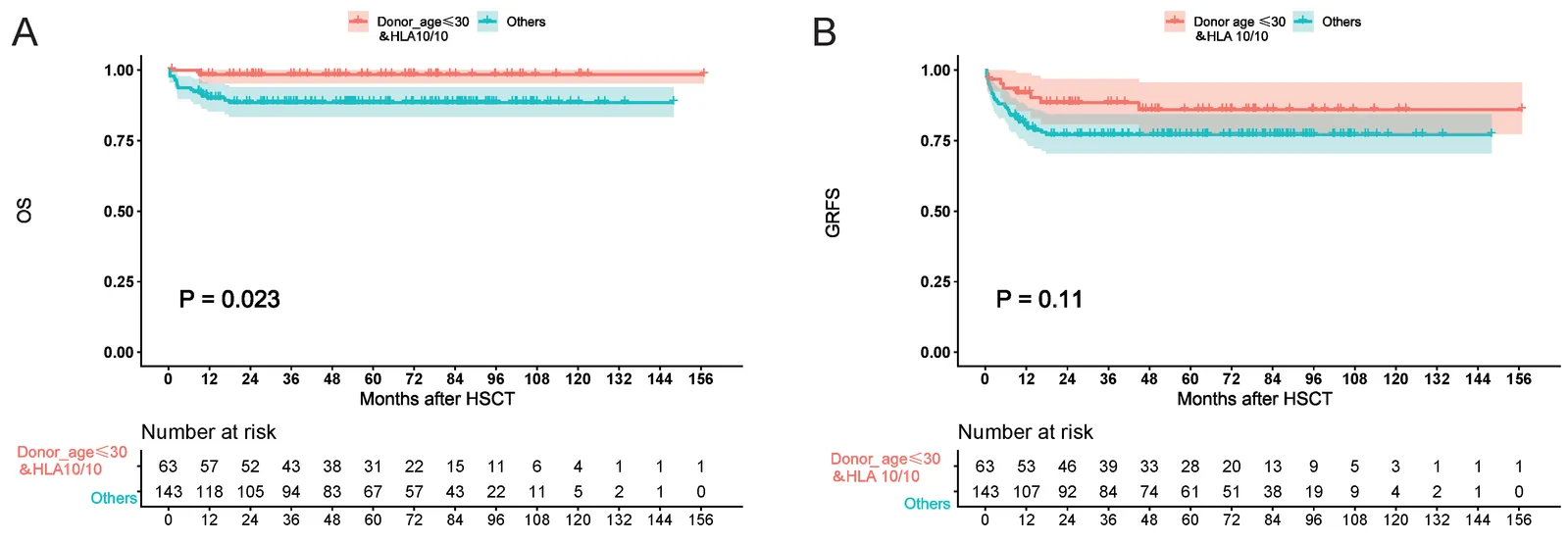
** OS (A) and GRFS (B) of patients with SAA**.

**Table 1:** Characteristic features of AA Patients With URD-HSCT(n=206).

Characteristic	
Age at transplantation, y, median (range)	28(5-64)
Age, y, n (%)	
≤30	118(57.3)
>30	88(42.7)
Sex, n (%)	
Male	110(53.4)
Female	96(46.6)
Diagnosis, n (%)	
NSAA	17(8.3)
SAA	143(69.4)
VSAA	46(22.3)
PNH clone	
Yes	46(22.3)
No	160(77.7)
Prior treatment, n (%)	
None/CSA ±TPO-RA	178(86.4)
ATG-based IST	28(13.6)
HCT-CI score, n (%)	
0	157(76.3)
1	38(18.4)
≥2	11(5.3)
HLA match, n (%)	
10/10	115(55.8)
9/10	91 (44.2)
ABO match, n (%)	
Matched	80(38.8)
Minor mismatched	51(24.8)
Major mismatched	50(24.3)
Different	25(12.1)
Donor recipient sex match, n (%)	
Female-female	14(6.8)
Female-male	19(9.2)
Male-female	82(39.8)
Male-male	91(44.2)
Mononuclear cell count, x10^8^ /kg, median (range)	9.9 (4.07-14.35)
CD34+ cell count, x10^6^ /kg, median (range)	4.87(1.05-19.8)
Neutrophil engraftment time, d, median (range)	12(9-22)
Platelet engraftment time, d, median (range)	12(8-55)

**Table 2:** Multivariable Analyses of Risk Factors for OS and GRFS in SAA patients.

Risk Factor	OS		GFRS	
	HR (95% CI)	*P*	HR (95% CI)	*P*
Donor age>30	**5.171(1.485-18.009)**	**0.010**	**2.210(1.153-4.235)**	**0.017**
Diagnosis_VSAA	0.845(0.212-3.363)	0.811	0.430(0.183-1.012)	0.053
HLA 9/10	**2.953(1.040-8.391)**	**0.042**	**2.274(1.198-4.316)**	**0.012**

## Data Availability

The datasets used and/or analyzed during the current study are available from the corresponding author on reasonable request.
