# Supplementary material for: Efficacy of Unrelated Allogeneic Hematopoietic Stem Cell Transplantation in Severe Aplastic Anemia: A Single-Center Retrospective Study
Source: Int J Med Sci. 2026 Sep 11;23(11):3313–22. doi: 10.7150/ijms.137416 (PMC13685043; doi:10.7150/ijms.137416)
Supplement: Supplementary file 1 [file ijmsv23p3313s1.pdf]

Supplementary Table 1. Clinical characteristics of SAA patients stratified by donor type.

Characteristic	Donor-Age≤30＆HLA 10/10 group(n=63)	Others group(n=143)	*P* value
Age at transplantation, y, median (range)	28(11-57)	28(5-64)	0.62
Age, y, n (%)			
≤30	40(63.5)	78(54.5)	0.23
＞30	23(36.5)	65(45.5)	
Sex, n (%)			
Female	32(50.8)	64(44.7)	0.42
Male	31(49.2)	79(55.3)	
Interval from diagnosis to HSCT, m, n (%)			**0.0002**
≤6	26(41.3)	99(69.2)	
＞6	37(58.7)	44(30.8)	
Diagnosis, n (%)			
NSAA	4(6.4)	13(9.1)	0.78
SAA	44(69.8)	99(69.2)	
VSAA	15(23.8)	31(21.7)	
Prior treatment, n (%)			0.25
None/CSA ±TPO-RA	57(90.5)	121(84.6)	
ATG-based IST	6(9.5)	22(15.4)	
HCT-CI score, n (%)			0.56
0	51(81.0)	106(74.1)	
1	9(14.3)	29(20.3)	
≥2	3(4.8)	8(5.6)	
Donor recipient sex match, n (%)			0.87
Female-female	5(7.9)	9(6.2)	
Female-male	5(7.9)	14(9.8)	
Male-female	27(42.9)	55(38.5)	
Male-male	26(41.3)	65(45.5)	
Mononuclear cell count, $x10^8$ /kg, median (range)	9.65(6.55-13.1)	9.92(4.07-14.35)	0.33
CD34+ cell count, $x10^6$ /kg, median (range)	5.38(1.53-14.32)	4.81(1.05-19.8)	0.47
